# Development of a Fluorescent Quenching Based High Throughput Assay to Screen for Calcineurin Inhibitors

**DOI:** 10.1371/journal.pone.0131297

**Published:** 2015-07-15

**Authors:** Abhisek Mukherjee, Kathleen Syeb, John Concannon, Keri Callegari, Claudio Soto, Marcie A. Glicksman

**Affiliations:** 1 Dept of Neurology, University of Texas Health Science Center, Houston, Texas, United States of America; 2 Laboratory for Drug Discovery in Neurodegeneration, Department of Neurology, Brigham and Women's Hospital and Harvard Medical School, Cambridge, Massachusetts, United States of America; Stanford University, UNITED STATES

## Abstract

Currently there is no effective treatment available for major neurodegenerative disorders associated to protein misfolding, including Alzheimer’s and Parkinson's disease. One of most promising therapeutic approaches under development focuses on inhibiting the misfolding and aggregation pathway. However, it is likely that by the time clinical symptoms appear, there is a large accumulation of misfolded aggregates and a very substantial damage to the brain. Thus, it seems that at the clinical stage of the disease it is necessary also to develop strategies aiming to prevent the neuronal damage produced by already formed misfolded aggregates. Chronic activation of calcineurin (CaN), a type IIB phosphatase, has been implicated as a pivotal molecule connecting synaptic loss and neuronal damage to protein misfolding. The fact that the crystal structure of CaN is also well established makes it an ideal target for drug discovery. CaN activity assays for High Throughput Screening (HTS) reported so far are based on absorbance. In this article we report the development of a fluorescent quenching based CaN activity assay suitable for robotic screening of large chemical libraries to find novel inhibitors. The assay yielded a Z score of 0.84 with coefficient of variance ≤ 15%. Our results also show that this assay can be used to identify CaN inhibitors with a wide range of potencies.

## Introduction

Neurodegenerative diseases (NDs) including Alzheimer’s Disease (AD), Parkinson’s Disease (PD), Transmissible Spongiform Encephalopathies (TSEs) or Prion Diseases and Huntington’s Disease (HD) are some of the most debilitating disorders, affecting abstract thinking, skilled movements, emotional feelings, cognition, memory, and other abilities. Synaptic abnormalities, neuronal death and accumulation of misfolded protein aggregates are the hallmark features of these diseases. Compelling evidence indicates that cerebral accumulation of at least one disease-specific misfolded and aggregated protein initiates the disease pathology [1,2]. Moreover, it seems that the mechanism by which misfolded aggregates are associated with neurodegeneration is similar among distinct diseases, and involves large changes of intracellular Ca^2+^ [3,4]. The rapid and sustained increase of Ca^2+^ in the cytoplasm after exposure to protein aggregates, produces hyper-activation of a key protein, termed calcineurin (CaN) [5,6]. CaN is the only Ca^2+^ dependent protein phosphatase present in neurons [7]. Optimum CaN activity is crucial for synaptic plasticity, memory and neuronal survival [8–10].

CaN is a Ca^2^+/Calmodulin (CM) dependent serine/threonine phosphatase present in all mammalian tissues and especially abundant in neurons [10–12]. It is a heterodimeric protein composed of a 61 KDa catalytic subunit (calcineurin A; CnA), and a 15 KDa regulatory subunit (calcineurin B; CnB) [10,13]. The catalytic core of CaN shares 41% and 39% amino acid sequence identity with Protein Phosphatase 1 and 2 (PP1 & PP2), respectively. However the three regulatory domains in the carboxy-terminal of subunit A distinguish CaN from others [10]. These domains are the CnB binding domain, the calmodulin-binding domain and the auto-inhibitory domain (CnAI). In resting conditions, the auto-inhibitory domain blocks the active site of the enzyme, resulting in very low activity. Ca^2+^-dependent binding of CM to the CnA-CnB complex removes CnAI from the catalytic site and activates the enzyme in a Ca^2+^ concentration dependent manner [13][14]. Optimum CaN activity is crucial to maintain the proper phosphorylation of different important targets in neurons [5,8,10,15]. However, chronically activated CaN shifts this balance towards the dephosphorylated state, severely affecting the cell. We and others have demonstrated disease-associated CaN hyper-activation in cultured cells, tissue slices and primary neuronal cultures upon exposure to misfolded proteins, such as aggregated Aβ (AD), α-synuclein, (PD), and prions (TSEs) [6,16–18]. Significantly higher CaN activity is reported in animal models of AD, TSEs, and HD and also in AD patients[6,19–21]. Phosphorylated cAMP response element binding protein (CREB), one of the CaN targets, induces expression of different proteins required for synaptic plasticity and neural growth [5,22]. Significantly lower levels of phosphorylated CREB, which cannot translocate to the nucleus to activate target genes, have also been reported in animal models of AD and TSEs [6,18] and in the hippocampus of AD patients [23]. Reduction of CaN activity was sufficient to rescue the phosphorylation state of CREB in mouse models of both pathologies, which leads to a striking improvement in memory and motor coordination [6,17,18,24]. Dephosphorylated by CaN, Nuclear Factor of Activated T cells (NFAT) c4 has been shown to be consistently present in the nucleus which is sufficient to produce dystrophic neurites and dendritic spine loss, the two most important morphological abnormalities of neurons reported in neurodegenerative diseases [25]. Consistent with this information, significantly higher nuclear localization of NFAT 3 has been observed in the hippocampus of AD patients which is co-related with the Mini-Mental State Exam scores (MMSE) for AD patients [26]. Hyper-activated CaN is not only associated to synaptic alterations, but it is also suggested to induce neuronal apoptosis through dephosphorylation of BAD in AD and TSEs [27]. Interestingly proper the phosphorylation state can be recovered by pharmacological reduction of CaN activity, in a mouse model of AD [18]. In fact, our own data indicate that pharmacological reduction of CaN activity not only rescues the behavioral phenotype, it also significantly increases the lifespan in a mouse model of TSEs [6].

The dual involvement of CaN on synaptic modulation and neural death (two of the main problems in NDs) makes it an ideal candidate for therapeutic intervention in NDs associated with protein misfolding. FDA approved CaN inhibitors, FK506 and cyclosporine (CyA), are currently used to prevent the immune response after organ transplants [13,28]. Neither of these drugs bind directly to CaN. Instead they bind to their cognate immunophilins, FKBP12 (for FK506) and cyclophilin (for CyA) respectively. The resulting complexes bind to CaN, inhibiting the substrate entry to the active site. This inhibition results in suppression of both humoral and cellular immune responses. However, long-term use of FK506 or CyA is reported to produce undesirable side effects, including nephrotoxicity, hypertension, hypercholesterolemia (for CyA only), diabetes and tremors (for FK506 only). Some of these side effects are attributed to the drug binding to immunophilins [29,30]. This is the major limitation for using these drugs (FK506 and CyA) for the treatment of neurodegenerative diseases. In addition, low penetration across the blood-brain barrier [31] requires large doses of these drugs to be administered in animals in order to observe therapeutic effects in the brain, dangerously increasing the extent and severity of undesirable side effects. Therefore, the search for a specific inhibitor for CaN seems promising as a feasible therapy. CaN inhibition assays developed so far for HTS are absorbance based [32,33]. Although absorbance assays are simple and robust, the reading depends on the path length, requiring higher assay volume even in a 384 well format. Thus the cost of screening/well increases seriously limiting the use of this assay in HTS. On the other hand, fluorescent assays are more sensitive and work well with volumes <10 μL, which is ideal for an HTS assay. Here, we describe the development of a robust high-throughput fluorescent quenching based assay to screen for small molecule inhibitors for CaN as well as the results of a pilot screen using this assay.

## Methods

### Reagents

Recombinant human calcineurin (Cat No. 539568), calmodulin (Cat No.208694), and enodthall (Cat No. 324760) were purchased from EMD Millipore. Substrate RII Phosphopeptide was purchased from American Peptide (Product No. 310258). Developing reagent Malachite green was purchased from BioAssay System, (Cat No. POMG-25H). The rest of the chemical reagents including HEPES, DTT, MnCl_2_, CaCl_2_ were all purchased from Sigma. White proxi-plates were purchased from PerkinElmer (Cat No. 6008280).

### Enzyme Assay

Enzyme master mix was prepared in 2X assay buffer containing 100 mM HEPES, 1 mM DTT,1 mM CaCl_2_, 2mM MnCl_2_. CaN and CM were incubated in equimolar ratio (if not otherwise mentioned) for 30 minutes at room temperature. Equal volumes of master mix and substrate were mixed to initiate the reaction. Reaction was stopped by adding malachite green reagent after 40 minutes. Absorbance of the end-product was measured at 620 nm using Molecular Devices’ Gemini Spectromax spectrophotometer. For enzyme characterization, reaction volume was 50 μL and malachite green volume was 40 μL. For the HTS assay, reaction volume was 5μL and malachite green was 2.5μL if not otherwise mentioned. Substrate was prepared by dissolving in RIIP MilliQ water. For K_m_ determination, different concentrations of RIIP were prepared by serial dilution of 4 mM stock in Mili Q water For the pH titration, instead of HEPES a mixture of 50mM MES, 100mM Tris was used. The buffer was adjusted to different pH using acetic acid. The equation used to fit K_m_ determination curve and CM titration curve was the following:

$$v=\frac{v_{max}\left[s\right]}{\left(K_{m}+\left[s\right]\left(1+\frac{\left[s\right]}{K_{i}}\right)\right)}$$ (1)

Where: *v* = initial velocity, *v*_*max*_ = maximum velocity, [*s*] = substrate concentration, *K*_*i*_ = substrate inhibition constant.

$$Y=\frac{B_{max}{\left[CM\right]}^{h}}{\left(IC_{50}^{h}+{\left[CM\right]}^{h}\right)}$$ (2)

Where: *B*_*max*_ = Maximum CaN activity, [*CM*] = CM concentration. *h* = Hill cofactor.

### Miniaturization of Assay

Colorimetric CaN activity was converted to fluorescence quenching based assay by modifying a technique, previously described by Zuck and colleagues [34]. Quenching of background fluorescence from white proxi-plates (Perkin Elmer, cat# 6008280) by phospho-malachite green complex was determined. We first tested the maximum background fluorescence emission of white-proxi plates at 610nm by exciting the plate at different excitation wavelengths using Gemini SpectroMax EM fluorimeter. Finally, we chose excitation at 573nm since it produced maximum emission at 610nm. We also observed that maximum emission changes depending on the plate types used. In the presence of different assay solutions, the plate was excited at 573nm and fluorescence was measured at 610nm. Samples quenched the intrinsic fluorescence of the plate. The highest concentration of phospho-malachite green quenched the most. Malachite green control with no phosphate quenched the least. The fluorescence output from the quenching assay was converted into OD using the following equation to prepare the phosphate standard curve:

$$OD=-\text{ log}\left(\frac{signal\;from\;test\;well}{average\;signal\;from\;blank}\right)$$ (3)

[34]

### HTS assay

The HTS assay was designed in a similar way as described by Glicksman and colleagues before [35]. 384-well v-bottom polypropylene plates (Perkin Elmer) containing 0.4 μL of compound at a concentration of 1.67 mM in DMSO in columns 1–22 or DMSO alone in 23, 24 were used as compound source plate. 13 μL of assay buffer was added using a Multidrop Combi liquid dispenser (Thermo Scientific), resulting in an intermediate concentration of 50 μM for each compound. Enzyme master mixture (5nM CaN and 5nM CM), substrate (250μM RIIP) and malachite green were transferred to respective 384 source plates (15μl/well). This volume was calculated for 4 assay plates. 1 μL of compound solution was transferred from the compound source plate to the assay plate (Perkin Elmer proxiplates,cat# 6008280) using the Biomek NX automated liquid handling system with a 384-channel pipetting head (Beckman-Coulter, Brea, CA) followed by addition of 2ul of enzyme master mixture in column 1–23 and only assay buffer in column 24. The mixture was incubated for 30 minute at room temperature. The reaction was initiated by adding 2μL substrate using a Biomek NX automated liquid handling system from the substrate source plate. Final compound concentration in the assay was 10μM and DMSO 0.6%. The reaction was terminated by adding 2.5μL of malachite green after 1 hour using Biomek NX automated liquid handling system and the fluorescence was measured using Geminai Spectromax fluorometer (ex:573nm; em:610nm).

### Inhibition Assay

For the fluorescence quenching based inhibition assay, 1.86 mg of endothall (EMD; cat # 324760) was dissolved in 1 ml MilliQ water to prepare a 10 mM stock. The stock solution was serially diluted to 9.5 μM in MilliQ water. 1 μL from each stock was incubated with 2μL of the master mix for 30 minutes followed by the enzyme reaction initiated by 2μL substrate addition. For confirmation of hits does response inhibition was measured in 96 well absorbance based format, using the same conditions as mentioned in the enzyme assay section. Stock solutions of the candidates were prepared in DMSO. Serial dilutions of the compounds were prepared in 1X assay buffer keeping the DMSO concentration constant at 0.5%. 20μL of 2X enzyme master mix was incubated with 10μL of the compounds at different concentrations for 30 minute at room temperature. The reaction was initiated by the addition of 20μL of 500μM substrate. The reaction was stopped after 40 minutes by addition of 40μL malachite green reagent and the absorbance was measured as previously described. Percentage of inhibition was calculated using the following formula.

$$\%\;inhibition=\frac{100\left(positive\;control-test\;well\right)}{positive\;control}$$ (4)

*IC*_50_ was determined using the following formula using Prizm software.

$$Y=bottom+\frac{\left(top-bottom\right)}{\left(1+10^{\left(\text{log}IC_{50}-x\right)hill\;slope}\right)}$$ (5)

### Compound library

The compound library screened consisted of approximately 1400 small molecules, including compounds approved by the Food and Drug Administration (FDA), a purified natural products library, and compounds purchased from Maybridge Plc. (Cornwall, UK), Bionet Research Ltd. (Cornwall, UK), Prestwick (Illkirch, France), and ChemBridge (San Diego, CA). Non-FDA approved compounds were selected from the different vendors by applying a series of filters, including for clogP and predicted solubility. All small molecules tested generally adhere to Lipinski’s rules (i.e., molecular weight <500, H-bond donors ≥5, H-bond acceptors ≥10, and logP<5) and contain a low proportion of known toxicophores (i.e., Michael acceptors and alkylating agents) and unwanted functionalities (i.e., imines, thiol, and quaternary amines) and have been optimized to maximize molecular diversity. Compounds for HTS were stored as DMSO stocks.

## Results

### Characterization of calcineurin enzyme activity

**Fig 1:**
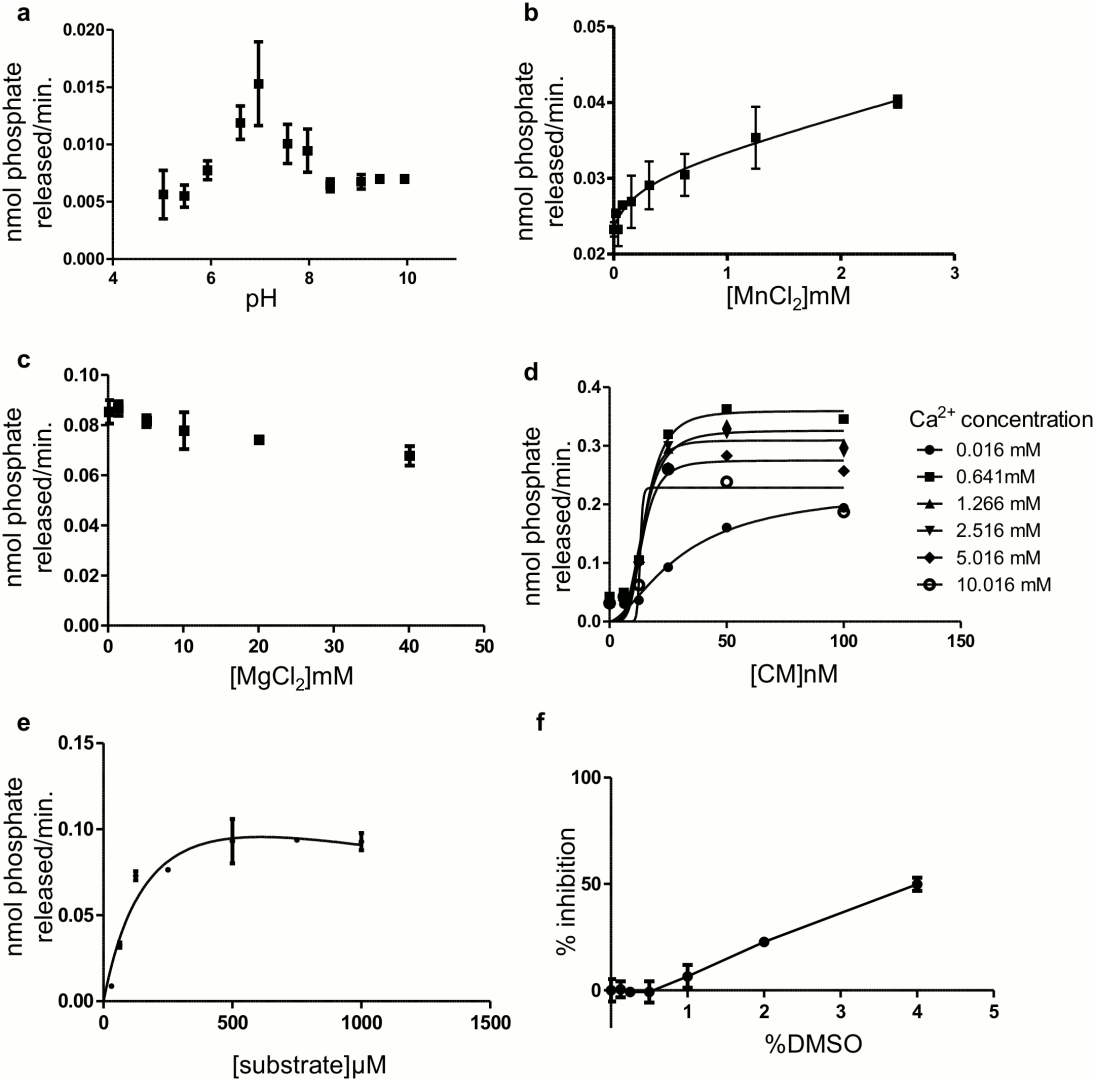
Characterization of enzyme activity. (a) In order to test the effect of pH on CaN activity, CaN assay was performed at different pH. Our data suggested that pH 7 is optimum for CaN activity. To study the effect of bivalent metal ions on CaN activity, enzyme activity assays were performed at different MnCl_2_ (b) and MgCl_2_ (c) concentrations. The data indicated that bivalent manganese increased CaN activity almost two times (b) whereas magnesium had not effect (c). Note that (a), (b) and (c) were performed in absence of CM. (d) The concentration of CM which produces 50% activation (IC_50_ CM) of CaN was determined by plotting initial reaction velocities at different Ca^2+^ and CM concentrations. Our data indicated that CM produces half maximum CaN activity at 1:1 molecular ratio. (e) Enzyme K_m_ was determined by plotting initial velocity at different substrate (RIIP) concentrations. The K_m_ was 213 μM. (f) DMOS tolerance of the enzyme was assayed by performing the assay at different DMSO concentration ranging from, 0.125 to 4%. Our data indicate that at ≤0.5% DMSO did not alter enzyme activity. The assays were done in duplicate (except for experiment in Fig 1d) and the data were expressed as means and standard error. The fittings were done using Prism software.

The active subunits of calcineurin is encoded by three different genes Aα, Aβ and Aγ. Aα is predominantly expressed in brain [36,37]. Therefore, we purchased recombinant human CaN, comprising CnAα and CnB subunits. A 19 amino acid peptide fragment (81-99aa), phosphorylated at the serine residue, from regulatory subunit type II of the CaN substrate cAMP-dependent protein kinase (PKA) was used as the substrate [33]. This peptide fragment, called RIIP, is a very well-established substrate for *in vitro* CaN activity assay. Initially, we determined the optimal buffer composition for CaN activity. Enzyme activity was tested at pH values ranging from 5 to 10 in 0.4-unit intervals, identifying an optimal pH of 7.0 (Fig 1a). It has been reported that there is a binuclear metal center in the active site of CaN which binds to negatively charged phosphate ions during the catalysis [38]. Therefore, we tested the effect of the bivalent metal ions Mg^2+^ and Mn^2+^. Our results suggest that Mn^2+^ almost doubled the activity of activation (Fig 1b), whereas the addition of Mg^2+^ (the enzyme stock already has 6mM) does not produce any effect (Fig 1c). However, we have not used more than 1mM MnCl_2_ in the assay to avoid precipitation. CaN-CM binding is required for CaN activation. Since this activation step is unique for CaN compared to other phosphatases, we decided to target both CaN-CM binding site together with the catalytic site. However, saturating concentrations of CM in the assay might mask the effect of a weak CaN-CM inhibitor. Thus, Ca^2+^ and CM were first titrated simultaneously against CaN to determine the ratio of CaN/CM required for 50% enzyme activity. Our Ca^2+^/CM cross-titration study indicated that a molar ratio of 1:1 (CaN:CM) produces 50% activation of CaN in the presence of 500μM Ca^2+^ (Fig 1d). Thus, all experiments were carried out at 1:1 molar ratio of CaN/CM. Finally, we determined the K_m_ value of CaN, using phosphorylated RII peptide as substrate (RIIP) (Fig 1e). The K_m_ value estimated under our experimental conditions was 213 μM, which is very similar to previously published results [38]. However, we did observe significant substrate inhibition, which was not previously reported. All of the compounds in the chemical library are solubilized in DMSO. Once all assay parameters were characterized, we performed a DMSO tolerance test for CaN assay, which indicated the enzyme activity, is not affected by ≤0.5% and there is less than 5% inhibition at ≤1% DMSO(Fig 1f).

### Miniaturization of the assay in 384 well format

After detailed enzyme characterization and standardization of reaction conditions, we converted our malachite green based absorbance assay into a fluorescent quenching based assay using a method previously described by Zuck and colleagues [34]. Maximum background fluorescence emission of white 384 wells Perkin Elmer proxy plates at 610 nm was determined by exciting the plate at different wavelengths (data not shown). The developing reagent malachite green, upon binding inorganic phosphate, produces an intense green color, which absorbs at 610–620 nm. Thus, fluorescence emission of the plate is effectively absorbed by phospho-malachite green complex. This quenching of fluorescence emission of the plate at 610 nm was used for detection of phospho-malachite green complex. Next, the reading was converted into OD using a previously described formula [34].

**Fig 2:**
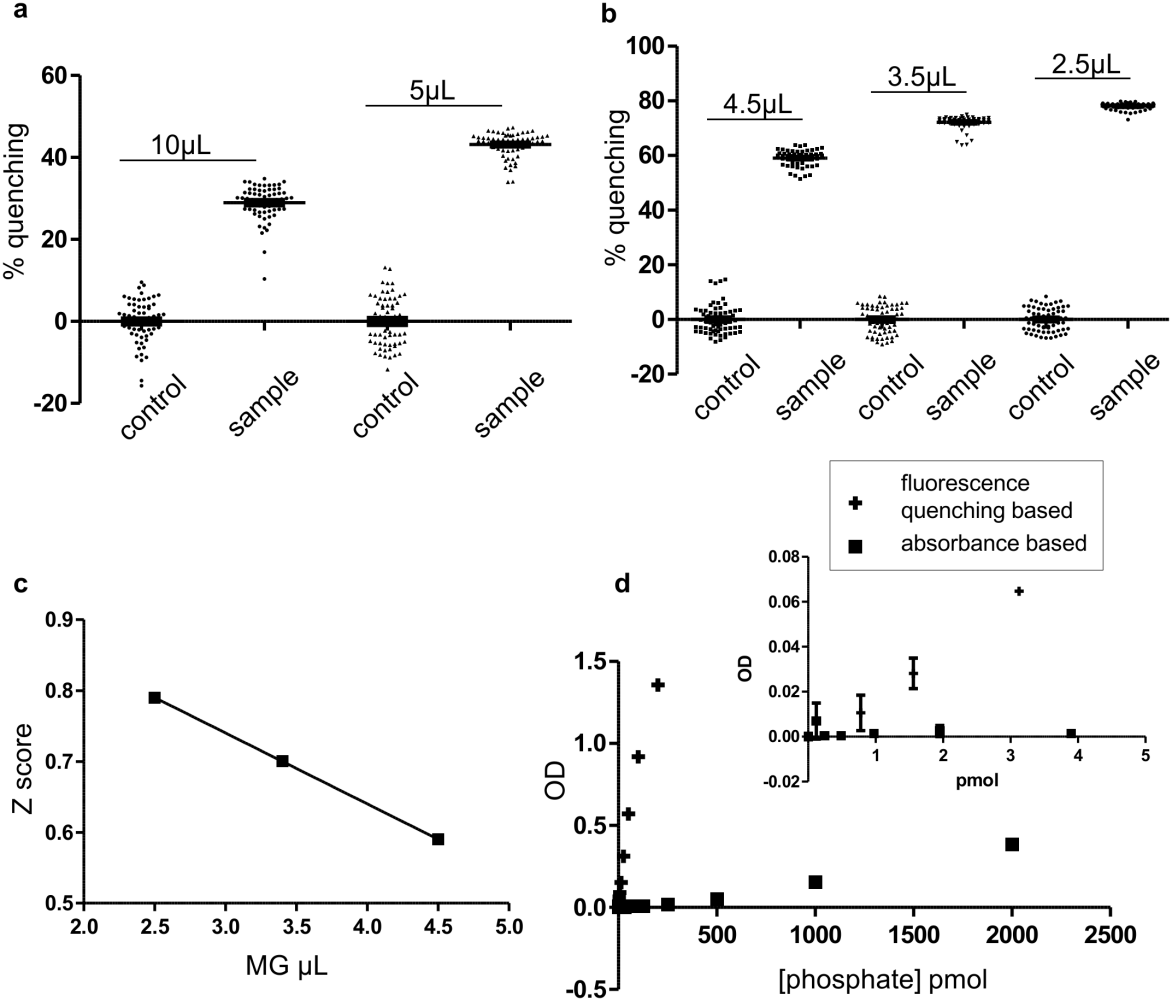
Miniaturization of the assay using phosphate standard. (a) The effect of different volumes of standard phosphate solution was studied by adding it into 384 Perkin Elmer white proxy plates. The malachite green volume was kept at ratio of 1.25:1 (reaction volume: developing reagent volume). (b) The influence of the malachite green quantity was measured using 200pmol phosphate in 5 μL volume. (c) Z score was plotted against malachite green volume. Z score linearly decreases with malachite green volume. (d) A phosphate standard curve was generated using white plate fluorescence quenching versus absorbance assay in 96 well plates. Inset shows high sensitivity phosphate detection at lower concentrations. Our data indicate that the 5μL assay volume with 2.5μL developing reagent produces the optimum result using white proxy plates. In panels a and b, raw data was converted to OD and then % of quenching was calculated.

Reaction volume was titrated using a phosphate standard solution keeping concentration constant (40 μM). Our data indicated that sensitivity increased with the reduction of the volume, with 5μL reaction volume producing the highest Z score of 0.38 (64 replicates in each group) (Fig 2a). Although the Z score was not optimum yet we deliberately did not reduce the reaction volume beyond 5μL since it is difficult for our robotic system to handle low volumes accurately. Instead, we titrated the volume of the developing reagent malachite green, keeping the reaction volume constant at 5μL. Sensitivity was increased by lowering the developing reagent volume (Fig 2b). In fact, linear increase of Z score was observed with decreasing malachite green volume finally yielding a Z score of 0.79 (64 replicates in each group) with 2.5μL (Fig 2c). After standardizing the assay format, a phosphate standard curve was prepared (Fig 2d). Our data indicated that this assay is highly sensitive at low phosphate concentrations (0.78–200 pmol), which cannot be achieved using a standard absorbance assay that has a detection limit of 31.25 pmol (Fig 2d).

### Validation of the fluorescence-quenching assay

**Fig 3:**
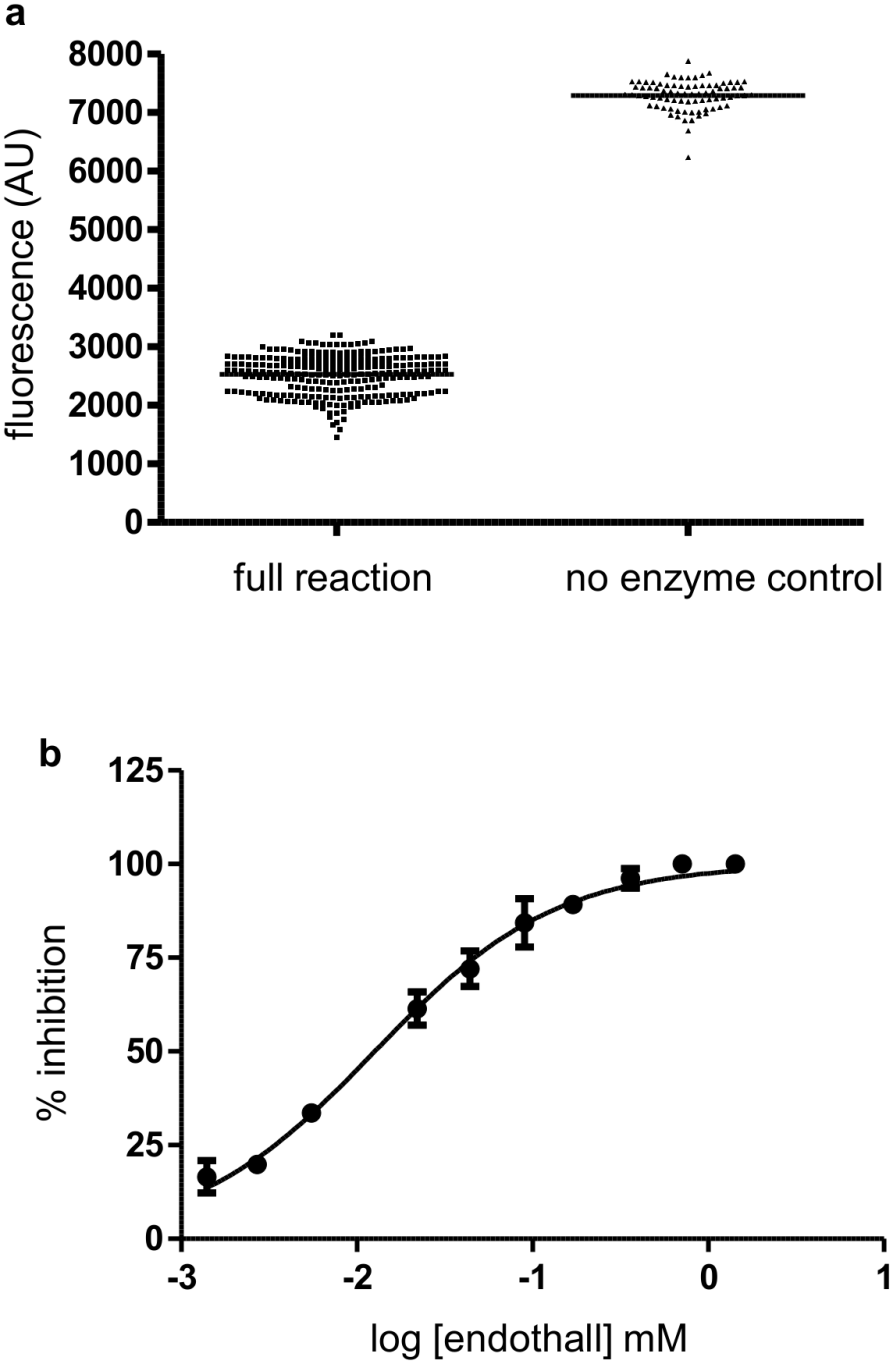
Validation of the assay. White plate fluorescence assay was validated using CaN and a known inhibitor. (a) Complete enzyme assay was performed in 272 replicates (full reaction) and compared with 80 replicates of no enzyme control. Assay was done manually in 384 well plates. The assay yielded a Z score of 0.63. (b) Endothall, a known inhibitor of CaN, was used to validate the assay format. The assay was done in duplicate and the data point represents mean and standard error. IC_50_ was calculated by prism software, using Eq 5, was 12.63μM.

Once the technique was optimized using inorganic phosphate standards, we performed the CaN phosphatase assay using previously optimized parameters with RIIP as a substrate. The enzyme concentration and reaction time were meticulously determined to keep the product concentration within the linear range of the detection. Using 272 replicates of full reaction and 80 replicates of no enzyme controls we have obtained a Z score of 0.63 (Fig 3a). To study further the fluorescence quenching assay format, we have tested endothall, a known inhibitor of CaN. The IC_50_ of endothall determined by the fluorescence quenching assay was 12.63μM (Fig 3b), which was very similar to previously published values [39], further validating the assay format.

### Automation of the assay and hit generation

**Fig 4:**
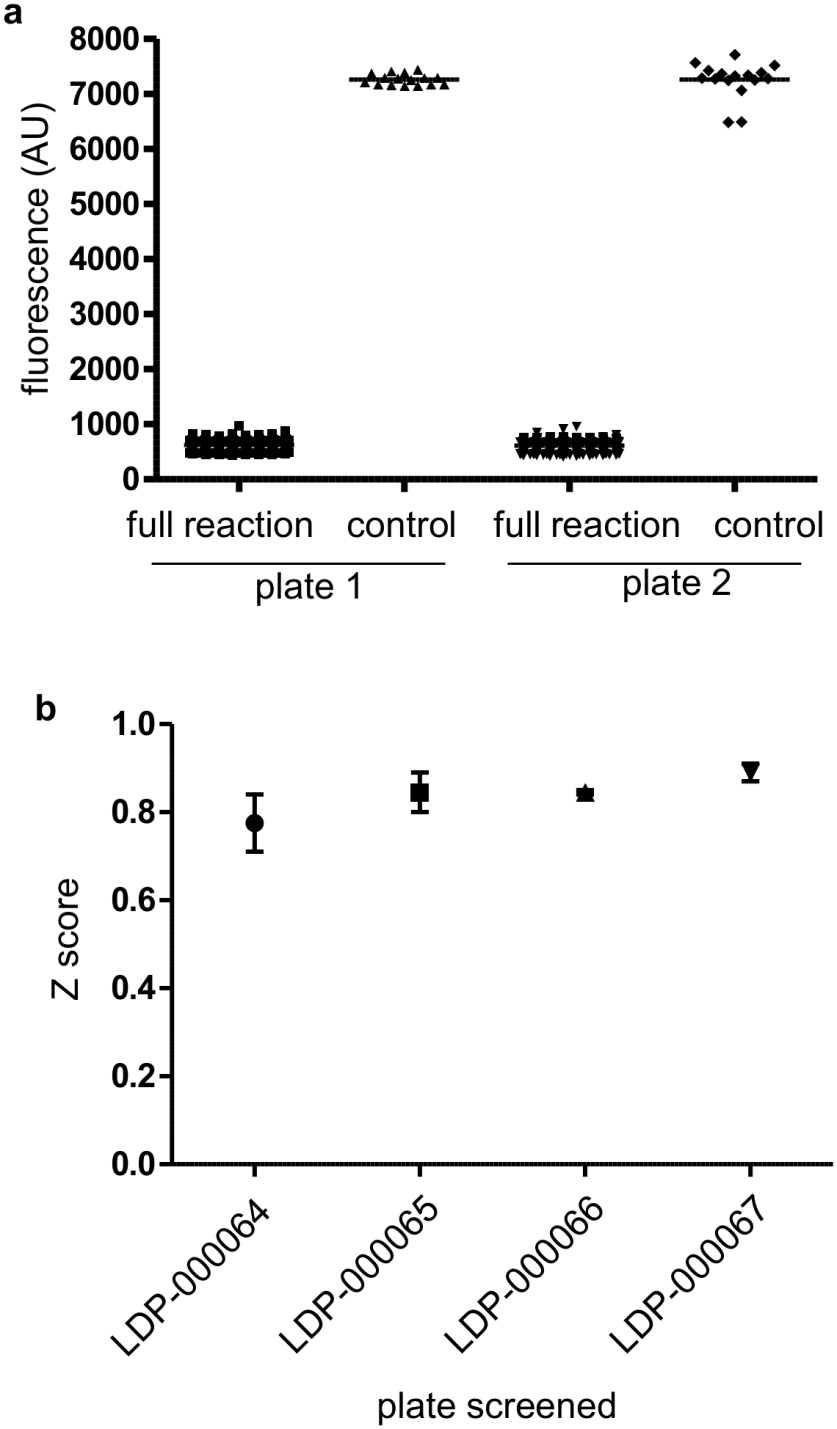
Automation and screening. The complete assay was automated and performed in a similar way mimicking all steps of the actual screening. 100% DMSO was used to replace the compound. (a) Two full 384 plates were used. Plate 1 and 2 yielded a Z score of 0.91 and 0.81; % CV 13.99 and 15.12 for the full reaction, respectively. (b) Z scores of 4 pilot plates screened in duplicate using this automated assay format.

**Fig 5:**
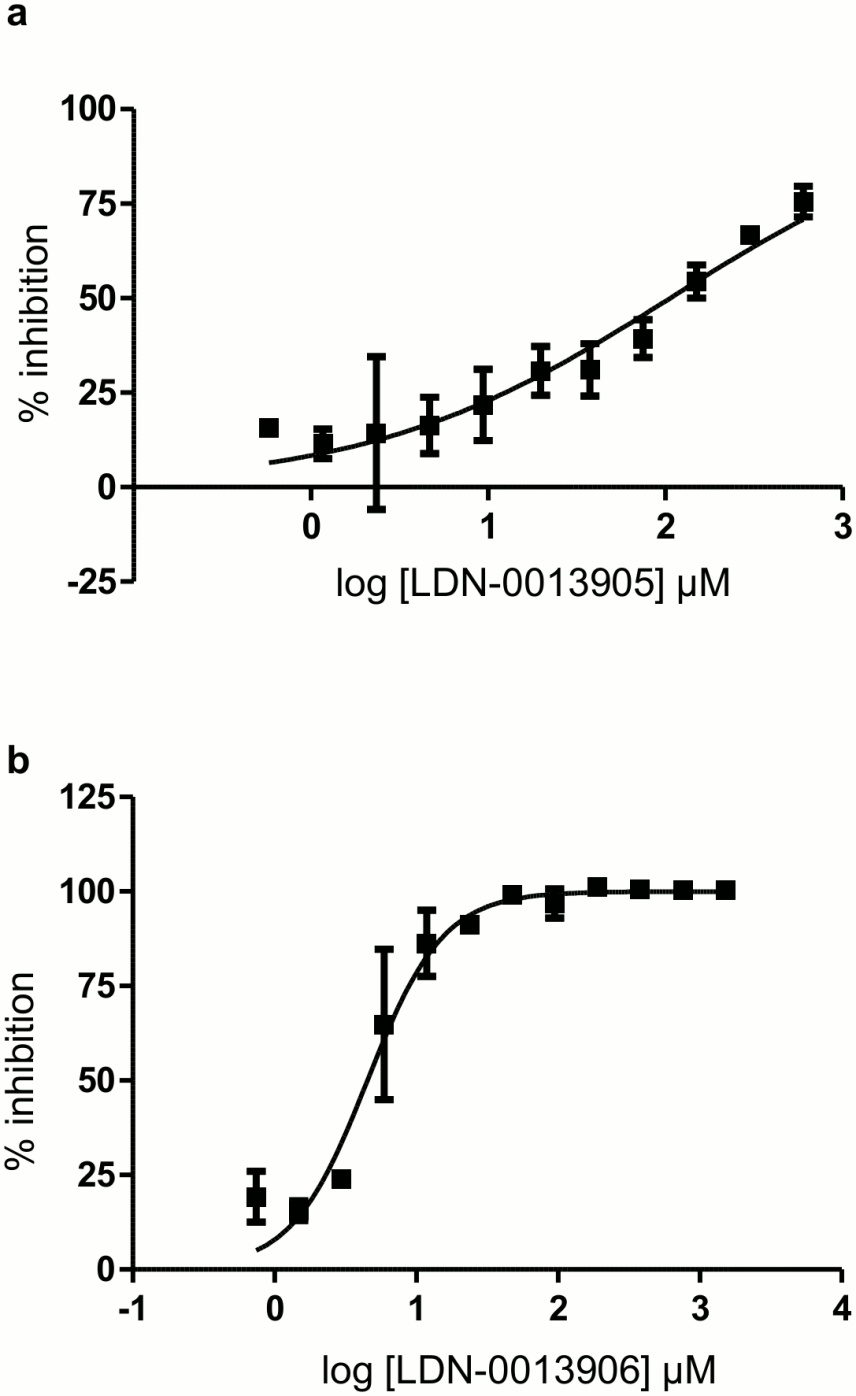
Validation of hits. Approximately 1400 compounds were screened, in duplicate, against CaN using this assay format. 50% inhibition was set as the threshold to identify hits. Out of 16 hits identified, 4 were selected on the basis of Lipinski's rule and commercial availability. Two of the selected compounds produced a dose-dependent inhibition and at least more than 50% inhibition. The reaction was done in duplicate and the data point represents mean and standard error. The IC_50_ were determined using Prism software, using Eq 5, and the values were (a) 106.4 μM for LDN-0013905 (flunarizine hydrochloride), (b) 4.5 μM for LDN-0013906 (tri-fluoperazine dihydrochloride). The fact that tri-fluoperazine dihydrochloride (LDN-0013906) is a known inhibitor of CaN further validated our assay format.

**Fig 6:**
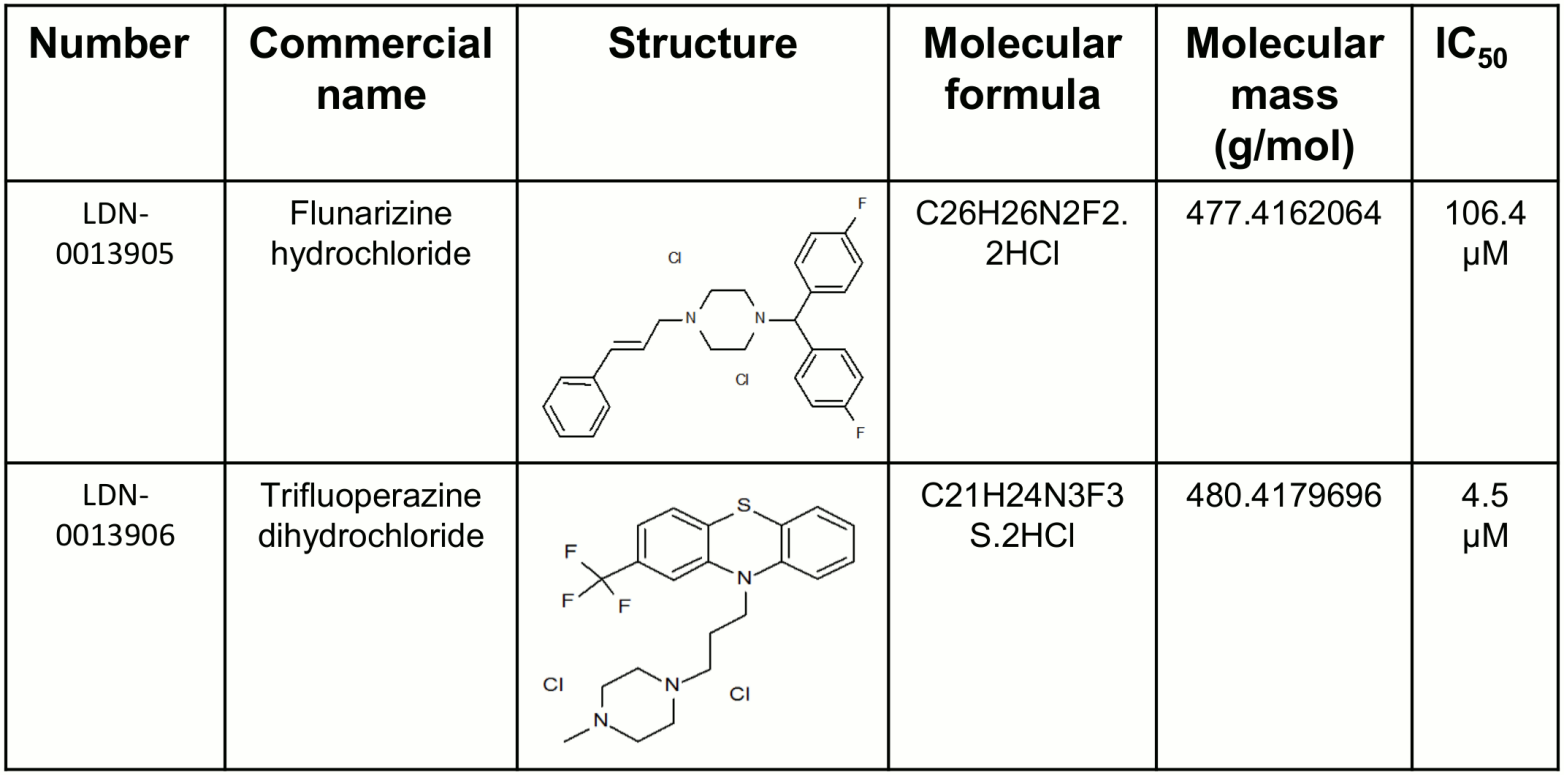
Chemical structure and formula of identified hits. Chemical structures, masses and formulas of the inhibitors are listed in the tabular format.

Next the assay was automated by mimicking all the liquid handling steps required for the HTS, including reagent transfer from different source plates. 100% DMSO was used instead of the compounds for standardization of the robotic handling. Enzyme concentration was titrated using automated reagent handling in order to maximize the signal/background ratio staying within the linear range of phosphate detection (data not shown). Next, two full plates were tested at the determined concentration of the enzyme (7.5nM). The automated assay yielded a Z’ score of 0.91 and 0.81 with %CV of 14 and 15 for plate 1 and plate 2, respectively (Fig 4a). Since the automated assay fulfilled all the statistical criteria for HTS we performed a pilot screen of 4 plates (about 1400 compounds tested at 10 μM final concentration) in duplicate. The Z’ score of the duplicate plates were similar and ranged from 0.71 to 0.91 (Fig 4b). We used a generous threshold of 50% inhibition to identify low potency inhibitors. The screening resulted in 16 hits from the 1400 compounds tested, producing a hit rate of 1.14%. Next, these 4 compounds were tested directly by using a 12 point dose-response using freshly purchased stock from a commercial vendor. We selected these 4 compounds based on commercial availability. Intrinsic fluorescence of the molecules can produce false positive results in our florescence quenching assay format. Therefore we have used regular 96 well format absorbance based CaN assay to confirm the hits. 2 of these 4 compounds demonstrated moderate activity with ≥50% inhibition in a confirmatory assay. The IC_50_ values calculated from Prism software, using a.4 parameter fit, were in the micro molar range (Fig 5a and 5b, and Fig 6) which is typical of hits identified from HTS. The chemical structure and formula of the hits are shown in (Fig 6). Compound LDN-0013906 (tri-fluoperazine dihydrochloride), had an IC_50_ of 4.5μM and was a previously identified CaN inhibitor [39,40] further validating the assay for HTS.

## Discussion and Conclusion

Understanding the pathways by which misfolded proteins cause neurodegeneration and disease is essential to develop much needed efficient treatments for NDs. Recent exciting data in various NDs have implicated hyper-activation of CaN in the cellular pathways leading to synaptic loss and neuronal death [5,6,16,18,41]. Strikingly, administration of CaN inhibitors to animal models of AD and TSEs appear to have therapeutic benefits [5]. The crystal structure of calcineurin has been solved which should be useful for structure-activity relationship studies and lead optimization processes. Therefore, CaN appears to be a promising drugable target against NDs. Previously, CaN inhibition assays developed for so far for HTS were absorbance based and lacked sensitivity [32,33]. In this article, we describe the development of a highly reproducible, sensitive, and robust fluorescent read-out based assay for CaN activity which is compatible with high throughput robotic screening.

We characterized the enzyme in our settings using a 96 well format and very carefully optimized the effect with different conditions. Finally, we were able to convert an absorbance based phosphatase assay into a highly sensitive, low volume, fluorescence read out based assay compatible with HTS. Although the concept of white plate fluorescent quenching was previously described, it is not widely used for HTS screening, most likely due to some technical limitations. In our study, we have addressed these limitations [42]. Deep yellow Malachite green detection reagent itself quenches the background fluorescence of the plate in a dose-dependent manner (data not shown). Although the quenching by malachite green alone is much lower compared to the green phosphomolybdate complex, it may interfere with the sensitivity of the assay. By carefully titrating the volume of malachite green we were able to detect as little as 780 fmol phosphate which is more than 30 times lower than the previously detected quantity [34]. Although very sensitive compare to the absorbance assay, in the low phosphate concentration range (0.78–200 pmol) the assay completely saturates at 300 pmol phosphate. Therefore, caution has to be taken while determining the enzyme concentration and the reaction time. We have shown, by scrupulous calculation of enzyme concentration and reaction times that even 12 point dose-responses can be performed using this format which reproduces the IC_50_ value of a known inhibitor, endothall. The technique depends on the background fluorescence of the assay plate material. However, by detailed characterization of the assay plate, we were able to ensure reliability, which is reflected by the Z’ score of 0.85 and % CV≤15 of the automated assay. Any fluorescent compounds with excitation/emission wavelength close to 573/610nm are expected to produce false positives. However, use of a different detection assay as confirmation of the hits (the absorbance based read-out), can easily rule out these possible false positive candidates. Confirmed hits generated from our assay have IC_50_ values ranging from 4.5–106.4 μM indicating that this platform can be used to screen inhibitors with wide range of potencies. The fact that one of the hits generated by our screening platform was a previously known CaN inhibitor further validated the HTS assay. Our novel and highly reproducible, inexpensive and sensitive HTS assay for identification of CaN inhibitors may be useful for the discovery and development of compounds with therapeutic potential for various diseases, including some of the most insidious NDs. Before therapeutic development, our early hits will still need to be further validated in additional assays.
